# Chicago Medical Society

**Published:** 1870-12

**Authors:** 


### CHICAGO MEDICAL SOCIETY.

*November 21, 1870.*

President, Dr. T. D. Fitch, in chair.

Dr. Reid reported a case of spurious pregnancy in a woman of 32 years. The patient had borne two children, the youngest nine years before, and was pleased at the idea of being pregnant. Her menstruation did not wholly cease, but decreased very much. The fact that women do sometimes menstruate when pregnant was explained to her. Her abdomen enlarged, and after about four months she felt, as she supposed, the movements of the child. Her general health was good, bowels regular, and mind tranquil. When her nine months arrived, she had no symptoms of approaching confinement. At ten months, Dr. R. was sent for, to determine the reason for the delay. He found her enlarged to the size usual at term, but, on examination, the uterus was found not enlarged, and he stated to the woman that she was not pregnant. This she would not believe, because she had felt, for months, distinctly, "the motions of the child," which were exactly like those experienced during former pregnancies.

There could be found no tumor of any circumscribed or well-defined character, in the abdomen; there was no fluctuation; but a distinct tympanitic sound could be heard on percussing over parts of the abdomen. There could be learned no general symptom that could indicate the existence of a tumor of any sort.

After a short time, she began slowly to decrease in size, and in a few more months, was little larger than normal. What was the cause of the enlargement?

Dr. Marguerat had seen a case somewhat resembling this. A very fleshy woman stopped menstruating, and began to enlarge. She was told by one or two physicians, whom she consulted, that she was not; yet she was herself certain of it, having felt, as she supposed, the motions of the child.

The Doctor was called suddenly to attend her, labor having, as it was supposed, begun. A large amount of blood was being discharged per vaginum, and nothing else, and there was no pregnancy, which divulged a mental element, that lent some explanation to the cause.

The President related a case, not unlike the last, of a woman 35 years old, who was childless, and exceedingly anxious to become a mother. Her menstruation ceased, her abdomen enlarged, and pregnancy was supposed to exist. After the usual time, she was taken with pain and discharges of blood. A homœophathic physician was called; he made an examination, and told the friends that labor was progressing, and that everything was "right." He remained with her all night; labor did not progress rapidly, and he left, and called later in the day, to find little progress had been made.

The result of the case was, that the bleeding continued for a number of days, the woman's abdomen decreased, and she has never been delivered of a child.

Dr. Reid reported a case of supposed ovarian tumor in a young woman. The enlargement on palpation and percussion manifested the usual signs of a unilocular tumor; it seemed to have a distinct attachment to the side of the uterus or the broad ligament. Yet, after a few months, it began to subside, and was now almost entirely gone.

Dr. Paoli had known of a case, where, after delivery, a tumor was found attached to the uterus, of the size of a fœtal head, which disappeared gradually, without treatment.

Dr. Marguerat detailed a case of the same character as that of Dr. Paoli's, only in this the tumor developed after delivery, not being detected at the confinement. It was attached to the left broad ligament. Six weeks after delivery, was as large as a child's head, but gave no evidence of fluid when percussed.

Under the use of friction and the internal administration of mercury, the growth disappeared.

Dr. Ferine had known of a case in the Cook County Hospital, in which an ovarian tumor was supposed to exist in a woman delivered only a few months before. A consultation was called, to determine the time of operating, when it was thought best to give the patient a cathartic. A large quantity of fecal matter, which had distended the colon, was discharged, and the tumor was gone.

---

Tincture of Erigeron as a Hemostatic.—A writer in the *Dental Cosmos* recommends tincture of erigeron as more efficacious than Monsel's Salt in arresting the hemorrhage following the extraction of teeth. We suppose the E. Canadense is the species employed—one of the most common and widely-diffused weeds in America.—*Richmond and Louisville Medical Journal.*

